# High-Intensity Interval Training Enhances the Positive Effect of Pentoxifylline on Lipid Profile and Inflammatory Markers in an Endometriosis Animal Model

**DOI:** 10.1155/bmri/6742953

**Published:** 2025-03-10

**Authors:** Zahra Salehpoor, Maryam Koushkie Jahromi

**Affiliations:** Department of Sports Sciences, School of Education and Psychology, Shiraz University, Shiraz, Iran

**Keywords:** endometriosis, high-intensity interval training, inflammation, lipid profile, pentoxifylline

## Abstract

**Background:** The relationship between endometriosis and cardiovascular disease (CVD) is well established. However, the effects of various exercise training modalities and the anti-inflammatory effects of pentoxifylline (PTX) remain inadequately understood. This investigation is aimed at evaluating the effects of PTX, both independently and in conjunction with high-intensity interval training (HIIT) and moderate-intensity continuous training (MICT), on lipid and inflammatory markers including triglycerides (TGs), total cholesterol (TC), high-density lipoprotein cholesterol (HDL-C), low-density lipoprotein cholesterol (LDL-C), and C-reactive protein (CRP) in a rat model of endometriosis.

**Materials and Methods:** Sprague–Dawley's rats were divided into two primary groups: the healthy control group that received no intervention and the induced endometriosis group. Endometriosis was surgically induced in rats, and those with confirmed endometriotic lesions were further categorized into six groups: control, MICT, drug of PTX (D), MICT+D, HIIT, and HIIT+D. Two weeks after laparotomy, PTX consumption and exercise training were performed for 8 weeks. PTX was administered orally at 100 mg/kg/day. MICT and HIIT sessions were conducted 5 days per week, with MICT beginning at 55% of maximum capacity for 31 min in the first week and progressing to 70% of maximum capacity for 46 min by the eighth week. HIIT sessions consisted of 2 min of running followed by 1 min of passive rest at 85% of maximum capacity, starting with seven intervals in the first week and increasing to twelve by the end of the eighth week. The macroscopic size of endometriosis lesions was measured, and cardiovascular risk factors, including hs-CRP, TC, TG, HDL-C, and LDL-C, were assessed in serum samples.

**Results:** The induction of endometriosis was associated with elevated cardiovascular risk factors, including hs-CRP, TC, and TG. HIIT+D significantly decreased lesion volume (*p* < 0.0001, 95%confidence interval (CI) = 57.239–94.718), hs-CRP (*p* = 0.049, CI = −54.083 to − 29.478), TC (*p* = 0.045, CI = −38.607 to − 25.392), and TG (*p* = 0.042, CI = 25.531–55.801). PTX significantly decreased lesion volume (*p* < 0.0001, CI =34.709–73.919) and TC (*p* = 0.016, CI = −45.153 to − 30.179).

**Conclusion:** All interventions except MICT reduced lesion volume, whereas only HIIT+PTX and PTX, in the order of importance, improved some cardiovascular risk indices in the rat model of endometriosis.

## 1. Introduction

The development of endometrial-like tissues, such as endometrial glands and stroma, outside of the uterus is linked to endometriosis [1]. Globally, 190 million women of reproductive age—nearly 10%—are afflicted by this chronic illness [2]. Endometriosis can be associated with various symptoms and diseases. One newly discovered area of interest in women's health is the relationship between endometriosis and subclinical atherosclerosis [3]. Previous findings have suggested an association between endometriosis and lipid profile [4] as well as an increased risk of developing cardiovascular disease (CVD) in various populations [5]. However, the underlying etiology is poorly understood despite its significant impact on public health. Atherosclerotic plaques [6] and endometriosis [7] share similar biological mechanisms such as chronic inflammation that underlie their development, as highlighted by some studies. This means that the pathophysiology of endometriosis may involve some of the same atherogenic risk factors, like dyslipidemia [8]. Dyslipidemia, which contributes to the development of atherosclerosis, is defined as an elevation of plasma levels of triglycerides (TGs), total cholesterol (TC), or both, or a low level of high-density lipoprotein (HDL) [9]. Endometriosis is linked to CVD [10] and may be explained by mechanisms including inflammation, endothelial dysfunction, and atherogenic lipid profiles [10]. The impact of endometriosis on atherosclerosis was investigated in a previous study using a murine model. The results indicated that women with endometriosis may be at higher risk of CVD because of cytokines related to inflammation released by these lesions [11].

According to a case-control study, women with endometriosis had significantly lower levels of high-density lipoprotein cholesterol (HDL-C) and significantly higher levels of TGs, TC, and low-density lipoprotein (LDL) cholesterol (LDL-C) in their serum than controls. Although women suffering from endometriosis show an elevation in all lipoproteins, the most remarkable lipoproteins are LDL and HDL levels [8]. LDL-C exerts various effects, such as anti-inflammatory and antithrombotic effects [12]. The LDL is of high clinical importance because, in the presence of inflammation and high oxidative stress, it is oxidized. Oxidized LDL then damages the endothelium, resulting in the accumulation of inflammatory cells. Oxidized LDL leads to systemic inflammation and the formation of atheroma in arteries. Higher levels of LDL in endometriosis women pose them with a higher risk of atheromatous plaque formation and atherosclerosis [13].

There is also a known relationship between inflammatory indices and atherosclerosis [10]. An inflammatory factor that significantly predicts the risk of CVD is high-sensitivity C-reactive protein (hs-CRP) [14]. As a sensitive biomarker of chronic inflammation [15], it may indicate subclinical inflammation in the serum of endometriosis-affected women [1]. Endothelial dysfunction–related all-cause mortality can be predicted by hs-CRP [3] which not only suggests a systemic inflammatory status but also potentially represents the onset of atherosclerosis [3, 9].

There are several pharmacological and nonpharmacological therapies for endometriosis and related symptoms. One of the most widely used pharmacological treatments is pentoxifylline (PTX) (3,7-dimethyl-1-(5-oxohexyl)-3,7-dihydro-1Hpurine-2,6-dione), a methyl–xanthine derivative and a nonselective phosphodiesterase (PDE) inhibitor with anti-inflammatory properties which is currently recommended for peripheral artery disease. Patients with atherosclerosis and coronary artery disease have reported significant modulating effects of this medication, according to clinical trials assessing its anti-inflammatory qualities [16]. Some studies have indicated that using PTX might delay the progression of atherosclerosis and decrease the likelihood of cardiovascular events, particularly by regulating the body's inflammatory response [17]. PTX can suppress the production of CRP as a marker of inflammation both in vitro and in vivo [18]. PTX's impact on endometriosis and related conditions, however, is a topic of debate [19]. Therefore, it appears that other complementary treatments, such as exercise training, in addition to PTX can be helpful in the treatment process.

Cardiovascular risk factors are decreased, and the lipid profile is improved with regular exercise [12, 20]. Because aerobic exercise training raises HDL-C by increasing lipoprotein lipase (LPL) concentration, it enhances blood lipid metabolism [12]. This type of exercise accelerates lipid transfer to cells, reduces serum TG [21, 22], and lowers TC levels and serum LDL-C [23].

Physical activity/exercise has also been proposed as a method of treating or preventing endometriosis as an inflammatory disease [24]. Different modes and intensities of aerobic exercise training are available, and studies have suggested that moderate-intensity continuous training (MICT) can be used to reduce inflammation [25]. Prolonged, low-to-moderate-intensity, acute, or chronic endurance training can increase HDL levels [12, 26].

Recently, it has been suggested that individuals with metabolic disorders who engage in high-intensity interval training (HIIT) might experience less inflammation [27]. Due to its effectiveness in reducing cardiometabolic disease risk factors, HIIT has become more popular [28]. HIIT and MICT may have varying effects on physiological functions. HIIT was found to be more effective than MICT in increasing HDL-C levels in younger adults, according to several studies [12, 29]. In addition, a significant decrease in TG was found following HIIT compared with MICT [28]. HIIT can control or reduce inflammatory factors in the condition of performing for a long period, whereas short-term HIIT may induce different effects and increase inflammation [30]. According to a systematic review, HIIT can reduce inflammatory biomarkers, such as CRP. Further studies are suggested to compare the effects of HIIT with MICT on the inflammatory profile [31]. MICT has also been considered an effective method for reducing the risk factors of CVD [32]. However, in recent years, HIIT has been suggested as the most effective exercise training for improving cardiorespiratory function and reducing CVD risk [33]. Some studies have been conducted to compare the effects of HIIT and MICT. An experimental study reported that HIIT was more effective than MICT in reducing the risks of CVD [34], while another study did not find this positive effect [35]. So, findings related to the effect of HIIT versus MICT on CVD risk factors are inconsistent [36]. Due to the relationship between inflammation and dyslipidemia [37] and the superiority of HIIT compared to MICT in reducing inflammation in cancer patients [38], it was hypothesized that HIIT can be superior to MICT in endometriosis. Also, due to the potential role of PTX as an anti-inflammatory drug in acute coronary syndrome [17], it was hypothesized that PTX can have a synergistic effect with exercise in cardiac risk factors. However, no study was found to compare the effect of HIIT and MICT as well as PTX and exercise on cardiovascular risk in endometriosis.

Available findings related to the interaction of exercise training and PTX indicated that horses treated with PTX had a greater decrease in Po2 values and a lesser increase in plasma lactate concentration during treadmill exercise. Also, the administration of PTX improved the RBC deformability of horses and exercise performance at rest and during treadmill exercise [39]. In addition, the positive effects of PTX administration were found on vascular conductance (VC) and left ventricular (LV) function at rest and during submaximal treadmill exercise in chronic heart failure (CHF) rats [40]. PTX administration improved LV function and elevated VC in CHF rats. These improvements were associated with PTX-induced reductions in plasma TNF-*α* variance [41]. Thus, this suggests that PTX administration could reduce iNOS and the systemic inflammatory response, which enhances local NO-mediated vasodilation via increased eNOS expression and function [42]. Therefore, it seems that the combination of exercise training on the treadmill and PTX can improve CVD function in disease conditions, which increases CVDs, such as endometriosis. The study hypothesized that HIIT will be more effective than MICT in improving lipid profiles and reducing inflammatory factors in rats with endometriosis, with additional benefits observed when combined with PTX, leading to greater reductions in inflammatory biomarkers compared to either method alone.

According to a recent review in 2023 [10], it is advised that future studies examine how preventive measures affect the risk of CVD in endometriosis-affected women. Therefore, using a model of endometriosis-ridden rats, this study assessed the effects of HIIT, MICT, and PTX, individually and in combination, on the lipid profile and inflammatory factor of hs-CRP.

## 2. Materials and Methods

### 2.1. Animals and General Procedures

Shiraz University of Medical Sciences (SUMS) Center of Comparative and Experimental Medicine provided 63 female Sprague–Dawley rats (8–9 weeks old, 220 ± 20 g) for this experiment. The animals had unrestricted access to food and water, and they were housed in a room with artificial 12-h light and 12-h dark cycles. The animals were maintained based on the guidelines for the use and care of laboratory animals set forth by the National Institutes of Health, Bethesda, and approved by the Research Ethics Committee of Shiraz University (IR.US.REC.1401.005).

Based on the number of groups and the statistical test, it was estimated that there were five animals in each group, [43] and eight rats were assigned to each group in the case of animal reduction. Daily vaginal smears were obtained and examined under a light microscope to identify the rats with normal estrous cycles. Three normal estrous cycle rats were split into two groups: (1) eight healthy rats were assigned to the healthy control (HC) group without any treatment; (2) 55 rats were used to induce endometriosis. To confirm endometriosis induction, a second look laparotomy was done 6 weeks after the initial procedure.

Eight weeks following initial surgery, 48 rats diagnosed with endometriosis were randomly assigned to six groups:

1. Control group: received oral gavage of saline for 8 weeks.
2. MICT group: engaged in MICT (5 days/week).
3. Drug of PTX(D) group: administered PTX (100 mg/kg/d) orally.
4. MICT+D group: combined MICT with PTX consumption.
5. HIIT group: engaged in HIIT.
6. HIIT+D group: combined HIIT with PTX consumption.

Training groups without drug consumption also received saline gavage to control for the gavage effect. Rats underwent a third laparotomy and were sacrificed, and samples were obtained for additional molecular analysis 8 weeks following medication and exercise training interventions to assess the impact of treatments on endometriotic lesions.

### 2.2. Induction of Endometriosis

Endometriosis was induced through surgery using the method defined by Vernon and Wilson [44] with a few revisions. Ketamine hydrochloride (10%; 100 mg/kg; Alfasan, Netherlands) and xylazine (2%; 10 mg/kg; Alfasan, Netherlands) were used to anesthetize the rats. Then, beginning 1 cm below the xiphoid and ending with a 4 cm midline incision, the rats' abdomen was opened. Both the cervical junction and uterotubal ends of the left horn of the uterus were cut and removed. A longitudinal cut was made to the uterine horn. Four round (5 × 5 mm) pieces of the distal uterine tissue were removed by punch biopsy and placed in a warm, sterile, 0.9% saline solution. On the left side of the peritoneal cavity, two implants were sutured with proline 5–0 in the areas of visible vasculature, with the endometrial surface facing the peritoneum. The abdominal muscles, fascia, and skin were then stitched. After applying oxytetracycline (OTC Spray, Iran) to the incision site, the animals were released from anesthesia. At this stage, three rats perished from hemorrhage. To confirm the successful induction of endometriosis, a follow-up laparotomy was conducted 6 weeks following the initial procedure, and the sites where the endometrial implants were transplanted and sutured were reviewed and examined. The viability of endometrial implants was verified in 48 rats by the observation of good and healthy vascular supply and the growth of implants. Of the 55 experimental rats that were left, 4 did not show any signs of endometriosis and were removed from the study because 3 of the rats died during the surgery. Eight weeks following the initial surgery, interventions involving medication and exercise training were carried out.

### 2.3. PTX Administration

PTX (Extended Release, Amin Laboratory, Esf, Iran) was administered orally every day for 8 weeks after endometrial tissue implantation. To determine the drug dose equivalency between humans and experimental animals, a translation formula based on a body surface area normalization method was used to calculate the dosages [45]. One hundred milligrams per kilogram per day of PTX was given to the animals in the PTX-consuming groups. The PTX consumption was modified based on the weekly weight changes of the rats.

### 2.4. Familiarization and Maximal Running Test

Eight weeks following endometriosis induction, each animal spent 3 days getting acclimated to the stop treadmill before spending 5 days getting acclimated to the moving treadmill, which ran at the speed of 10 m/min for 5 minutes each day. To ascertain each animal's training intensity, the animals in exercise groups then underwent three alternating days of maximum running tests. Leite et al. employed an incremental test. VO2max was calculated using a method defined by Amaral et al. The animals were placed on the stopped treadmill for 5 min before being allowed to run at a maximum speed of 10 m/min on the 10° incline treadmill. This process was repeated every 6 s. The speed was then increased by 3 m/min every 2 min until the animals became exhausted [46, 47]. This test was conducted in the first, fifth, and eighth weeks, to determine the level of intensity of exercise training programs.

### 2.5. Exercise Training Protocols

The exercise training protocols (Table 1) were started 48 h after the maximum running test. The participants worked out 5 days a week for 8 weeks on a motorized treadmill with an inclination of 10°. Based on the results of the maximum capacity test, the training intensity was modified based on the percentages of maximum (the speed at exhaustion time was considered a maximum capacity). Every group engaged in a 5-min warm-up at a speed of 10 m per minute, followed by a 5-min cool-down. The exercise training regimens were modified from earlier research [46, 47].

During the first week of the MICT, according to the maximal running test, the maximum speed was 30.9 m/min, and the speed was set at 55% of the maximum capacity or 17 m/min based on the relevant reference; the duration of training in the first week was set to 31 min. The speed increased progressively until the eighth week, so by the end of the eighth week, the speed was increased to 70% of the maximum capacity for 46 min. During the second week of the MICT, according to the maximal running test, the speed was set at 58% of the maximum capacity or the speed of 18 m/min, and the duration of training was 31 min. During the third week, according to the maximal running test, the speed was set at 64% of the maximum capacity or the speed of 20 m/min, and the duration of training was 37 min. During the fourth week, the speed was set at 67% of the maximum capacity or the speed of 21 m/min, and the duration of training was 40 min. In the fifth week, according to the maximal running test, the maximum speed was 38.09 m/min, the speed was set at 63% of the maximum capacity or the speed of 24 m/min, and the duration of training was 45 min. In the sixth week, the speed was set at 70% of the maximum capacity (70% of 38.09 m/min), so the obtained speed was 27 m/min, and the duration of training was 46 min. In the seventh week, the speed and duration of training were set the same as in the sixth week (27 m/min and 46 min). In the eighth week, according to the maximal running test, the maximum speed was 38.57 m/min, so the speed was set at 70% of the maximum capacity or the speed of 27 m/min, and the duration of training was 46 min.

Training sessions for the HIIT included 2 min of running and 1 min of passive rest. The exercise volume increased gradually over the weeks until reaching 12 periods with a speed of 85% maximum capacity. The first week's HIIT consisted of 7 periods at a speed of 85% of maximum capacity [46]. According to the maximal running test, the maximum speed was 28.23 m/min, so the speed was set at 85% of the maximal running test or 24 m/min, and the duration of training was 31 min. In the second week, HIIT consisted of 7 periods at a speed of 85% of maximum capacity. Thus, the obtained speed was 26 m/min, and the duration of training was 31 min. In the third week, HIIT consisted of nine periods at a speed of 85% of maximum capacity. Thus, the obtained speed was 27 m/min, and the duration of training was 37 min. In the fourth week, HIIT consisted of 10 periods at a speed of 85% of maximum capacity. Thus, the obtained speed was 30 m/min, and the duration of training was 40 min. In the fifth week, according to the maximal running test, the maximum speed was 37.64 m/min, and the speed was set at 85% of the maximal running test, so the obtained speed was 32 m/min, and the duration of training was 45 min. In the sixth week, HIIT consisted of 12 periods at a speed of 85% of maximum capacity. Thus, the obtained speed was 35 m/min, and the duration of training was 46 min. In the seventh week, HIIT consisted of 12 periods at a speed of 85% of maximum capacity. Thus, the obtained speed was 38 m/min, and the duration of training was 46 min. In the eighth week, according to the maximal running test, the maximum speed was 44.70 m/min. The speed was set at 85% of the maximal running test, so the obtained speed was 38 m/min, and the duration of training was 46 min.

### 2.6. Anesthetizing, Sampling, and Scarifying

Anesthesia was administered intramuscularly to all rats for 8 weeks following drug and exercise training interventions, before sampling, and scarification. The anesthesia mixture consisted of 10%ketamine hydrochloride (100 mg/kg, Alfasan, Netherlands) + 2%xylazine (10 mg/kg, Alfasan, Netherlands) + 1%acepromazine (1.5 mg/kg Alfasan, Netherlands). Once the rats were completely unconscious, the abdomens were opened by making a 4-cm midline incision beginning 1 cm below the xiphoid, and samples were collected. Every endometriotic lesion was meticulously measured for height, width, and length. The ellipsoid volume formulation (*π*/6 × length × width × height) was utilized to calculate the volume [48]. Rats were sacrificed by having their hearts stopped in diastole with an intraventricular injection of 100 mg/kg of KCl while the animals were under general anesthesia. The rats' carcasses were then buried in an appropriate location.

### 2.7. Blood Sampling and Enzyme-Linked Immunosorbent Assay (ELISA)

Following the completion of the treatment, all of the rats were sedated, and blood was extracted using a syringe to puncture the heart. The serum was then extracted and centrifuged for 10 min at 1000∗g to obtain it for factor analysis. Using commercial kits (Pars Azmoon, Tehran kit, Iran), the concentrations of serum TC, triacylglycerols (TG), HDL-C, and LDL-C were measured. The ELISA Kit (No: MBS2508830, Size: 96T/48T/24T) was used to measure the serum concentration of hs-CRP. The Sandwich-ELISA instruction was applied in this ELISA kit.

### 2.8. Statistical Analysis

The statistical analysis was performed using SPSS Inc.'s 23.0 software (Chicago, United States). At first, the data were checked for normality with the Shapiro–Wilk test. Normally distributed data were subjected to one-way ANOVA analysis. Levene's test was used to determine whether the error variance of the dependent variable is equal across groups. If homogeneity of variances was confirmed in Levene's test, Tukey's post hoc test was used to determine the differences between groups, and otherwise, the Games–Howell post hoc test was used. The effect size was expressed based on the eta squared (*ƞ*^2^) method; the results were displayed as mean ± SD. *p* ≤ 0.05 was chosen as the statistical significance threshold.

## 3. Results

### 3.1. The Effect of PTX and Exercise Training on the Levels of TC, TG, HDL-C, and LDL-C

#### 3.1.1. TC Levels

**Figure 1:**
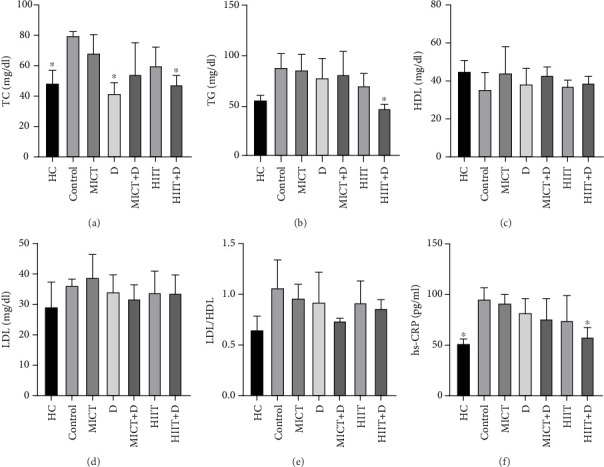
Comparison of TC, TG, HDL-C, LDL-C, and CRP between groups. (a) Comparison of TC (total cholesterol), (b) comparison of TG (triacylglycerols), (c) comparison of HDL-C (high-density lipoprotein cholesterol), (d) comparison of LDL-C (low-density lipoprotein cholesterol), (e) comparison of LDL-C/HDL-C ratio, and (f) comparison of hs-CRP (high-sensitivity C-reactive protein) in serum levels in different groups. HC, healthy control; MICT, moderate-intensity continuous training; D, drug (pentoxifylline); MICT+D, moderate-intensity continuous training+drug (pentoxifylline); HIIT, high-intensity interval training; HIIT+D, high-intensity interval training+drug (pentoxifylline). ⁣^∗^*p* < 0.05: significant difference compared to the control group. Data are presented as mean ± SD.

The results showed a significant difference in TC between groups (*p* = 0.014, *F* = 20.847, *ƞ*^2^ = 0.719). TC in the HC group was significantly lower than the control group (*p* = 0.04, 38.90%). Administration of D (*p* = 0.016, 47.28%, *ƞ*^2^ = 0.049, CI = −45.153 to − 30.179) and HIIT+D (*p* = 0.045, 40.17%, *ƞ*^2^ = 0.001, CI = −38.607 to − 25.392) reduced TC levels compared to the control group significantly; however, there was no significant difference in TC levels between MICT, D, MICT+D, HIIT, and HIIT+D groups (*p* > 0.05). In addition, MICT (*p* = 0.06, 28.77%, CI = −33.508 to −10.491), HIIT (*p* = 0.88, 18.88%, CI = −25.435 to 2.768), MICT+D (*p* = 0.99, 10.42%, CI = −28.118 to 16.785), HIIT+D (*p* > 0.99, −2.11%, CI = −8.701 to 10.701), and D (*p* = 0.99, −15.87%, CI = −3.440 to 16.773) interventions did not indicate significant differences with the HC group (Figure 1.

#### 3.1.2. TG Levels

The results showed a significant difference in TG between groups (*p* = 0.045, *F* = 14.373, *ƞ*^2^ = 0.638). TG in the HC group was nonsignificantly lower than the control group (*p* = 0.19, 44.59%, CI = 17.163–47.502). Administration of HIIT+D (*p* = 0.042, 45.86%, *ƞ*^2^ = 0.041CI = 25.531–55.801) reduced TG levels compared to the control group significantly. There was a nonsignificant decrease in TG levels in MICT (*p* = 1.000, 2.63%, *ƞ*^2^ = 0.357, CI = −17.155 to 21.821), MICT+D (*p* = 1.000, 8.27%, *ƞ*^2^ = 0.278, CI = −18.686 to 33.352), D (*p* = 0.926, 11.65%, *ƞ*^2^ = 0.230, CI = −12.330 to 32.997), and HIIT (*p* = 0.416, 20.30%, *ƞ*^2^ = 0.112, CI = 0.513–35.486) groups compared to the control group. Also, there was no significant difference in TG levels between the MICT, D, MICT+D, HIIT, and HIIT+D groups (*p* > 0.05). In addition, MICT (*p* = 0.26, 34.74%, CI = 13.249–46.750), MICT+D (*p* = 0.45, 30.73%, CI = 0.040–49.959), D (*p* = 0.58, 28.08, CI = 1.021–42.978), HIIT (*p* = 0.90, 20.28%, CI = 0.791–27.874), and especially HIIT+D (*p* = 0.99, −14.79%, CI = −15.30 to − 1.535) interventions did not indicate significant differences with the HC group (Figure 1b).

#### 3.1.3. HDL-C Levels

There was no significant difference in HDL-C between the groups (*p* = 0.759, *F* = 3.091, *ƞ*^2^ = 0.275). HDL-C in the HC group was nonsignificantly higher than in the control group (*p* = 0.25, 23.15%, CI = 0.223–18.443). Also, HDL-C levels were nonsignificantly higher in all groups compared to the control group. Respectively, higher HDL-C was observed in MICT (*p* = 0.775, 24.30%, *ƞ*^2^ = 0.001, CI = −0.443 to 17.776), MICT+D (*p* = 0.869, 20.56%, *ƞ*^2^ = 0.009, CI = −1.776 to 16.443), HIIT+D (*p* = 0.995, 9.35%, *ƞ*^2^ = 0.077, CI = −5.776 to 12.443), D (*p* = 0.998, 7.48%, *ƞ*^2^ = 0.094, CI = −6.443 to 11.776), and HIIT (*p* = 1.000, 4.67%, *ƞ*^2^ = 0.120, CI = −7.443 to 10.776) groups compared to the control group. However, the groups were not significantly different. In addition, HIIT (*p* = 0.88, 17.03%, CI = −16.776 to 1.443), D (*p* = 0.93, 14.81%, CI = −15.776 to 2.443), HIIT+D (*p* = 0.96, 13.33, CI = −15.109 to 3.109), especially MICT+D (*p* = 0.99, 4.44%, CI = −11.109 to 7.109), and MICT (*p* > 0.99, 1.48%, CI = −9.776 to 8.443) interventions indicated nonsignificant differences with HC group (Figure 1c).

#### 3.1.4. LDL-C Levels

The results showed no significant difference in LDL-C between the groups (*p* = 0.742, *F* = 3.484, *ƞ*^2^ = 0.299). LDL-C in the HC group was nonsignificantly lower than in the control group (*p* = 0.79, 21.32%, CI = −14.139 to 0.139). A nonsignificant decrease was observed in LDL-C levels in MICT+D (*p* = 0.933, 11.93%, *ƞ*^2^ = 0.026, CI = −11.473 to 2.806), HIIT+D (*p* = 0.991, 7.34%, *ƞ*^2^ = 0.066, CI = −9.806 to 4.473), HIIT (*p* = 0.995, 6.42%, *ƞ*^2^ = 0.076, CI = −9.473 to 4.806), and D (*p* = 0.998, 5.50%, *ƞ*^2^ = 0.086, CI = −9.139 to 5.139) groups compared to the control group; however, the administration of MICT (*p* = 0.991, 7.34%, *ƞ*^2^ = 0.261, CI = −4.473 to 9.806) increased LDL-C levels compared to the control group. In addition, D (*p* = 0.60, 14.55%, CI = −2.139 to 12.139), HIIT (*p* = 0.96, 13.72%, CI = −2.473 to 11.806), HIIT+D (*p* = 0.97, 12.87, CI = −2.806 to 11.473), and especially MICT+D (*p* = 0.99, 8.33%, CI = −4.473 to 9.806) interventions did not indicate significant differences with the HC (Figure 1d).

#### 3.1.5. LDL-C/HDL-C Ratio

The results showed there was no significant difference in the atherogenic index (LDL-C/HDL-C ratio) between the groups (*p* = 0.22, F =7.299, *ƞ*^2^ = 0.472). LDL-C/HDL-C ratio in the HC group was nonsignificantly lower than the control group (*p* = 0.19, 48.21%, CI = -0.714–-0.110). A nonsignificant decrease was observed in LDL-C/HDL-C ratio levels in MICT+D (*p* = 0.434, 35.69%, *ƞ*^2^ = 0.031, CI = −0.618 to − 0.023), HIIT + D (*p* = 0.844, 21.22%, *ƞ*^2^ = 0.144, CI = −0.500 to 0.094), HIIT (*p* = 0.961, 15.3%, *ƞ*^2^ = 0.215, CI = −0.472 to 0.182), D (*p* = 0.975, 13.45%, *ƞ*^2^ = 0.231, CI = −0.502 to 0.237), and MICT (*p* = 0.995, 9.66%, *ƞ*^2^ = 0.274, CI = −0.400 to 0.203) groups compared to the control group. In addition, MICT (*p* = 0.45, 38.50%, CI = 0.133–0.493), D (*p* = 0.58, 35.66%, CI = −0.034 to 0.593), HIIT (*p* = 0.63, 33.33%, CI = 0.023–0.511), HIIT+D (*p* = 0.82, 26.66, CI = 0.050–0.367), and especially MICT+D (*p* = 0.99, 12.95%, CI = −0.057 to 0.240) interventions did not indicate significant differences compared with the HC (Figure 1e).

### 3.2. The Effect of PTX and Exercise Training on the Levels of hs-CRP

The results showed significant differences in the CRP between the groups (*p* = 0.023, *F* = 21.653, *ƞ*^2^ = 0.726). CRP in the HC group was significantly lower than in the control group (*p* = 0.03, 58.55%, CI = −57.840 to − 37.493). Administration of HIIT + D (*p* = 0.049, 39.14%, *ƞ*^2^ = 0.024, CI = −54.083 to − 29.478) reduced CRP levels compared to the control group significantly. There was nonsignificant decrease in CRP levels in HIIT (*p* = 0.384, 25.06%, *ƞ*^2^ = 0.268, CI = −50.821 to 0.692), MICT+D (*p* = 0.440, 23.68%, *ƞ*^2^ = 0.291, CI = −44.912 to − 2.450), D (*p* = 0.707, 17.80%, *ƞ*^2^ = 0.390, CI = −33.492 to − 2.097), and MICT (*p* = 0.999, 4.78%, *ƞ*^2^ = 0.568, CI = −15.111 to 5.550) groups compared to the control group. However, there was no significant difference in CRP levels between MICT, D, MICT+D, HIIT, and HIIT+D groups (*p* > 0.05) (Figure 1f). In addition, MICT (*p* = 0.06, 42.78%, CI = 36.852–48.919), D (*p* = 0.21, 36.33%, CI = 15.163–44.580), MICT+D (*p* = 0.43, 31.42%, CI = 3.083–44.887), HIIT (*p* = 0.49, 30.16%, CI = −3.037 to 48.241), and especially HIIT+D (*p* = 0.99, 10.11%, CI = −4.272 to 16.045) interventions did not indicate significant differences compared to the HC group and were near to the HC group or healthy group.

## 4. Discussion

A recent publication from the study found that the treatments of D, MICT+D, HIIT, and HIIT+D significantly reduced the volume of endometriotic lesions compared to the control group. Within the treatment groups, the volume of lesions was not significantly different among the D, MICT+D, and HIIT groups, but all these groups showed a significant reduction in lesion volume compared to the MICT group. Notably, the HIIT+D group exhibited the most significant decrease in lesion volume compared to all other groups [49].

There have been considerable studies considering the link between subclinical atherosclerosis and endometriosis [3], whereas endometriosis-related chronic inflammation is thought to be the cause of CVD events in those who have the disease [50], endothelial dysfunction, and atherogenic lipid profile [5]. This is the first study that evaluated the effects of separate and combined PTX with MICT and HIIT, as therapeutic anti-inflammatory interventions on the systemic inflammation status and lipid profile in the rat model of endometriosis. Our results indicated that the endometriosis induction increased dyslipidemia factors of TC, TG, LDL-C, and atherogenic index (LDL-C/HDL-C) while decreasing HDL-C in the control (induced endometriosis) compared to the HC group, while HIIT+PTX and PTX by priority could improve lipid profile in endometriosis groups.

In terms of CRP levels, endometriosis was associated with an increase in this inflammatory marker in the control group compared to the healthy group. The combination of HIIT and PTX significantly reduced CRP levels, while other interventions—such as HIIT, MICT combined with PTX, PTX alone, and MICT—did not demonstrate significant effects compared to the control group.

Our study's findings demonstrated that, compared to the control group, PTX led to a nonsignificant drop in CRP levels (17.80%). In contrast to the nonsignificant findings of the present study, some previous studies investigated the anti-inflammatory effect of PTX in other diseases such as diabetes [51] and coronary artery disease [52]. In a randomized clinical trial, among patients on maintenance hemodialysis (HD), administration of PTX was associated with a substantial improvement of adequacy of dialysis and a significant prevention from serum CRP level increase [53]. A study found that the use of PTX in HD patients induced positive effects on inflammation and health-related QoL [54]. It has been proposed that PTX, a safe and well-tolerated medication, can stabilize plaque, slow the progression of atherosclerosis, and lower the risk of cardiovascular events [55]. The possible advantages of PTX in CV disorders have been reported by several clinical and experimental investigations [56]. In a brief review of the literature, in a study on animal subjects, PTX decreased the area of aortic atherosclerotic plaque and decreased CVD [57]. In another study, the PTX-treated cardiovascular event patients experienced significant reductions in serum C-reactive protein and TNF-*α* relative to the placebo group [58]. Goicoechea et al. [59] reported a significant decrease in markers of inflammation, such as CRP in patients with chronic kidney disease (CKD) who received PTX therapy. The primary enzyme involved in the production of proinflammatory cytokines is PDE [51]. It was expected that PTX's inhibition of PDEs leads to a decrease in inflammatory markers like hs-CRP [51]. A thorough review of the mechanisms by which PDE inhibitors, like PTX, can produce anti-inflammatory effects has already been published [52]. Specifically, it is probable that the suppression of the isozyme PDE 4 plays a significant role. PDE 4 is prominently present in inflammatory cells, such as endothelial cells [52, 60]. Therefore, it was expected that PTX with an inhibitory effect on these cells would lead to the reduction of inflammation and CVD risks in endometriosis rats. However, the nonsignificant findings may be attributed to the fact that endometriosis is not typically associated with a significant increase in CRP levels compared to other conditions, such as diabetes, COVID-19, or CVD. In our study, despite the nonsignificant reduction of CRP by PTX compared to the control group, it is somewhat near to the values of this factor in the healthy group which indicates the positive effect of this drug in improving the inflammatory factor of CRP. The significant role of PDE4 in the function of PTX represents a crucial limitation of this study due to the absence of measurements concerning this key factor. Future research should prioritize the assessment of PDE4 to better understand its impact on PTX function.

The results of our study showed that PTX led to a significant decrease in TC levels (47.28%); a nonsignificant decrease in TG (11.65%), LDL (5.5%), and LDL-C/HDL-C (35.66%); and finally, a nonsignificant increase in HDL (7.48%) compared to the control group. Cilostazol, a selective Type 3 PDE inhibitor, has been shown in earlier studies to raise HDL cholesterol and decrease serum TGs in diabetic rats by enhancing LPL activity. Findings revealed that increased cAMP stimulates the release of LPL from adipocytes, which hydrolyzes TGs in lipoproteins and may account for the drop in serum TG levels [61, 62]. It has been proved that PTX as an inhibitor of PDE effectively reduced TC, LDL-C, and TG, meanwhile increasing HDL-C in dyslipidemic rabbits [55]. Considering the relationship between lipid profile and inflammation, it seems that PTX with its anti-inflammatory effects can reduce lipid profile levels and prevent atherosclerosis in endometriosis. However, some other studies did not find any significant effect of PTX on lipid profile [9, 63]. Similar to our results, another study showed that PTX can reduce the lipid profile and be an effective treatment for patients with peripheral arterial disease with claudication [64]. Also, Ye et al. showed that PTX administration effectively alleviated the lipid profile and metabolic disorders [65]. In our study, despite the nonsignificant improvement of lipid profile by PTX (except for the significant reduction of TC) compared to the control group, it is somewhat near to the values of the healthy group which indicates the positive effect of this drug in improving lipid profile.

The impact of PTX on endometriosis and its symptoms remains difficult to understand, even though the effectiveness of the medication in treating endometriosis has been confirmed [19]. Therefore, it would appear that receiving PTX in addition to complementary therapies can enhance its benefits. Since their anti-inflammatory effects were well known, we used HIIT and MICT aerobic exercises as complementary interventions in our study for the first time.

We demonstrated that MICT (4.78%) and HIIT (25.06%) caused a nonsignificant decrease in CRP, although this effect was higher in the HIIT group. Exercise training has been shown to reduce the severity of systemic inflammation [66]. Regarding the effect of exercise training on decreasing CRP levels, exercise training has been suggested as an anti-inflammatory strategy [28, 67]. An identical effect on inflammation was not produced by different exercise training. After varying forms of exercise training, inflammatory mediators can be reduced through various mechanisms, such as an increase in skeletal muscle mass and immune cell adaptation [68], or a decrease in CRP levels [28, 67]. The effects of MICT on inflammation have been conflicting [28]. Jones et al. [69] showed that MICT did not cause significant changes in plasma levels of CRP [38]. In their study, Baygutalp et al. discovered that engaging in physical activity that leads to hypoxia can increase levels of CRP [70]. However, recent research findings did not show any changes in CRP levels in middle-aged men after engaging in MICT [30].

A unique, practical, and efficient training method, HIIT, has been used in multiple clinical studies to improve cardiometabolic health [71]. However, some studies reported no changes in CRP with HIIT [28, 72]. Some experimental studies reported HIIT was more effective than MICT in reducing risks of CVD [34], which was in contradiction with some other findings [35, 36]. The reason for the difference between the findings and nonsignificant findings of the study is probably due to the difference in the type of disease, the study population, and the duration of exercise training interventions.

We demonstrated that MICT and HIIT caused a nonsignificant decrease in TC (MICT = 11.30%, HIIT = 24.69%), TG (MICT = 2.63%, HIIT = 20.30%), and LDL-C/HDL-C (HIIT = 15.3% and MICT = 9.66%), although this effect was higher in the HIIT group. Thus, the results of our study indicated that MICT and HIIT statistically did not have a significant effect on lipid profile. Maintaining a normal lipid profile and lowering cardiovascular risk are thought to be dependent on regular exercise [12, 66, 73]. It has been suggested that training, especially aerobic exercise training, may have the potential to improve and increase the atheroprotective functions and plasma levels of HDL [12, 73] and 8–14 weeks of aerobic exercise improved TC, HDL-C, LDL-C, and TG [12, 71, 74–76].

Numerous studies demonstrated that moderate-intensity training improved lipid profile levels [26], and some research findings indicate that brief bursts of intense exercise training seem to have minimal effects on fat metabolism and have no discernible influence on lipid levels [26, 77]. The European Society of Cardiology (ESC) review found that there is no definitive evidence on the specific amount of exercise needed to enhance lipid profile and lower cardiovascular risk in both normolipidemic individuals and hyperlipidemic patients [12, 78].

Studies have shown that changes in inflammatory and lipid profiles are affected by the duration, volume, and especially the intensity of the training [79]. A major finding of the studies is that MICT significantly reduced proinflammatory cytokines and modified serum lipid profile [67, 75, 80]. In several conditions, including CVD, HIIT has recently become a competitive alternative [81], while providing equal or better health benefits compared to the popular exercise method known as MICT [72]. Elmer et al. found that after 8 weeks of HIIT, there was a statistically significant drop in TGs in HIIT as opposed to MICT [82]. However, some studies have not reported a difference between the two types of training. Wood et al. reported that although there was no discernible difference in the lipid profile effects between HIIT and MICT, HIIT was effective on HDL [29]. Fisher et al. [83] reported a significant difference between HIIT and MICT in reducing lipid profiles. Some studies have not found much of an effect of HIIT on cholesterol [28].

The precise processes by which exercise training improves the lipid profile are not known yet. Exercise has been shown to increase skeletal muscle's capacity to use fats rather than glycogen, which lowers lipid levels [84]. Exercise seems to be linked to increased levels of LCAT, an enzyme that facilitates ester transfer from HDL cholesterol [85]. After exercise training, greater LCAT levels have been reported [12]. Among other mechanisms, the effect of exercise activity on changing the lipid profile is the increase in gene expression of ABCA1-binding transporters [86] which causes the activation of the reverse transporter of cholesterol, the formation of HDL, and protection against atherosclerosis [79, 87]. LPL's primary function is to bind to most tissues' capillary endothelium to metabolize TGs in the circulatory system. This process generates free fatty acids which are absorbed subsequently by peripheral tissues. The majority of research indicates that LPL enzyme expression and activity in plasma heparin are elevated by aerobic exercise. Skeletal muscle is another area where lipolysis releases fatty acids that can be used as an energy source. Adipose tissue is another area where LPL activity liberates fatty acids. Exercise generally causes a decrease in adipose tissue LPL and an increase in skeletal muscle LPL activity, according to research on animals. However, studies on humans have shown that exercise also causes increases in LPL activity in both tissues [88]. So, a reason for controversies between findings may be animal or human subjects of the studies.

According to the findings of our investigation, HIIT increased the effects of PTX on TC, TG, and CRP factors, and the HIIT+D group indicated a significant decrease in TC (40.17%), TG (45.86%), LDLC/HDL/C (12.95%), and CRP (41.78%). Performing MICT and HIIT combined with PTX consumption in both groups caused nonsignificant positive increasing effects on HDL and a decreasing effect on LDL. Therefore, performing HIIT and consuming PTX separately and in combination could improve indices of CVDs in endometriosis. According to the underlying mechanisms mentioned for HIIT and PTX in previous sections of discussion, PTX and HIIT may induce synergistic effects on their related common mechanisms such as the inhibitory effect on PDEs, raised cAMP, increased LPL, and decreased iNOS, and as a result, it causes a decrease in BF and a decrease in systemic inflammation [62, 63], as well as the inhibitory effect on immune and inflammatory cells including endothelial cells [61, 89].

In our study, the intervention of HIIT alone and in combination with PTX improved factors, which were simulated with the healthy group, which could be due to the improvement of the endometriosis disease in our study groups or the overall effect of medication and exercise training and needs to be clarified in future studies.

The present study had some limitations, including the effect of exercise and PTX on cardiac risk factors, which was not assessed in the healthy groups; other risk factors were not measured which could be considered in future studies; the sample size was small, although it is adequate to ensure a sufficiently secure test power for judging our hypothesis; the information about the stage of the disease in the animal sample was not sufficient, which means that we could not estimate the improvement in the stage of the disease; also, heart tissue was not evaluated and can be investigated in endometriosis samples in future studies. Besides, other exercise training methods along with PTX, as well as longer periods of exercise training and medication, are suggested to be investigated in the future.

## 5. Conclusions

Our study has shown for the first time that the application of HIIT+PTX and PTX in the order of importance induces beneficial effects on reducing inflammation and lipid profile in rat models of endometriosis, and since inflammation and lipid profile are strong predictors of CVDs, by reducing these factors following the HIIT+PTX and PTX interventions, the probability of CVDs decreased in endometriosis. PTX alone or combined with MICT did not produce the expected benefits. Therefore, it can also be concluded that HIIT enhances the benefits of PTX in preventing atherosclerosis in endometriosis, highlighting their potential therapeutic benefits in this context.

## Figures and Tables

**Table 1:** Exercise training protocol.

**Training**	**Variable**	**Measure**	**W1**	**W2**	**W3**	**W4**	**W5**	**W6**	**W7**	**W8**
MICT	Frequency	Days	5	5	5	5	5	5	5	6
	Inclination	Degrees	10	10	10	10	10	10	10	10
	Velocity	Meter/minute	17	18	20	21	24	27	27	27
	Duration	Minutes	31	31	37	40	45	46	46	46
	Intensity	Continuous and progressive exercise 55~70% max capacity + constant								

HIIT	Frequency	Days	5	5	5	5	5	5	5	6
	Inclination	Degrees	10	10	10	10	10	10	10	10
	Velocity	Meter/minute	24	26	27	30	32	35	38	38
	Duration	Minutes	31	31	37	40	45	46	46	46
	Cycles	—	7	7	9	10	10	12	12	12
	Intensity	Cycles of 2 min progressive exercise ~85% max capacity + 1 min rest								

Abbreviations: HIIT, high-intensity interval training; MICT, moderate-intensity continuous training; W1–W8, Weeks 1–8.

## Data Availability

The data will be made available upon request.
